# Cardiorespiratory fitness in adolescence and premature mortality: widespread bias identified using negative control outcomes and sibling comparisons

**DOI:** 10.1093/eurjpc/zwaf267

**Published:** 2025-05-15

**Authors:** Marcel Ballin, Anna Nordström, Peter Nordström, Viktor Hugo Ahlqvist

**Affiliations:** Department of Public Health and Caring Sciences, Clinical Geriatrics, Uppsala University, Uppsala SE-75103, Sweden; Department of Medical Sciences, Rehabilitation Medicine, Uppsala University, Uppsala, Sweden; School of Sport Sciences, UiT The Arctic University of Norway, Tromsø, Norway; Department of Public Health and Caring Sciences, Clinical Geriatrics, Uppsala University, Uppsala SE-75103, Sweden; Department of Public Health and Caring Sciences, Clinical Geriatrics, Uppsala University, Uppsala SE-75103, Sweden; Department of Biomedicine, Aarhus University, Aarhus, Denmark; Institute of Environmental Medicine, Karolinska Institutet, Stockholm, Sweden

The global epidemic of physical inactivity, particularly among young people, is of grave concern for public health, and the importance of promoting physical activity and exercise for preventing premature morbidity and mortality has long been echoed throughout national and international guidelines.^1^ More recently, youth cardiorespiratory fitness—a marker reflecting both regular exercise and genetic predisposition—is gaining attention as a critical marker of health.^2,3^ In a scientific statement, the American Heart Association emphasized the importance of public health initiatives and policies targeting fitness in youth as this would be expected to yield substantial health benefits, quoting observational evidence suggesting lower mortality, including from cancer and cardiovascular causes.^2^ However, the extent to which these associations are causal remains uncertain, especially as the few existing randomized trials of clinical endpoints have rarely shown much benefit.^4^ One explanation for this discrepancy is that highly active and fit individuals are often different from their peers (e.g. large behavioural differences, socioeconomic resources, and genetics), and it can be challenging to eliminate all such factors in traditional observational studies.

To obtain more valid observational estimates, one could use methods that attempt to account for unobserved confounders. One such approach is negative control outcome analysis,^5^ where the association between fitness and an outcome not expected to be causally affected is examined (e.g. random accidents). An association between fitness and a negative control outcome could provide evidence of residual bias. Another approach that directly controls for unobserved confounders is sibling-comparison analysis, which accounts for all factors shared between siblings, including behavioural, environmental, and genetic confounders.^6^

In this study, we analysed a historical cohort of nearly all adolescent men in Sweden tested for cardiorespiratory fitness across several decades and followed these prospectively for premature all-cause, cancer, and cardiovascular disease mortality. To gauge the validity of the observational associations, we modelled the risk of accidental mortality (e.g. drownings, homicides, car accidents, etc.) as a negative control outcome, and we compared full siblings to account for unobserved familial confounding.

This study was approved by the Swedish Ethical Review Authority. The study population was obtained from the Swedish Military Service Conscription Register and included all men who participated in military conscription examinations between 1972 and 1995, during which conscription around the age of 18 years was mandatory for all Swedish men (∼90% coverage).^7^ At conscription, cardiorespiratory fitness was objectively assessed according to a standardized protocol using a maximal ergometer bicycle test, with test results recorded as Watt-max (Wmax).^7^ To this, we added data about full siblings from the Multi-Generation Register,^8^ and prospectively followed the cohort for all-cause, cancer, cardiovascular disease, and accidental mortality using the National Cause of Death Register (see Supplementary material online, *Table S1*).^9^

Participants were followed from the date of conscription until death, emigration, or 31 December 2023, whichever came first. To estimate cause-specific mortality, we censored individuals at the time of death from causes other than the one under study (e.g. in the cardiovascular mortality analysis, individuals were censored at cancer death). Flexible parametric survival models, extended to a marginalized between-within model in the sibling cohort, were used to estimate cause-specific hazard ratios (HRs) for the outcomes by quintiles of fitness, using age as the underlying time scale.^10,11^ Differences in HRs for the negative control outcome compared to the corresponding deciles for the primary outcomes were formally tested using cross-model Wald tests. Using these models, we also computed the standardized cumulative failure (net risk) and the cumulative incidence functions (crude risk) of cancer, cardiovascular disease, and accidental mortality, accounting for death from other causes than the one under study.^12^ All models were adjusted for age at conscription, year of conscription, body mass index at conscription, as well as for parental income and education level obtained from registers of Statistics Sweden. *P*-values <0.05 were considered statistically significant.

A total of 1 124 049 men with a mean age of 18.3 years at baseline, including 477 453 full siblings [excluding 10% with missing fitness or covariate data, or with extreme values (<100 Watt-max, body mass index ≤15 and ≥60 kg/m^2^)], were included (see Supplementary material online, *Table S2*). These were followed until a median (interquartile range) age of 56.6 (50.8 to 62.1) years (maximum 73.5), during which 64 911 deaths were recorded, of which 16 789 were cancer-related, 20 981 were cardiovascular-related, and 14 422 were accident-related (see Supplementary material online, *Table S3*).

Compared to the bottom quintile of cardiorespiratory fitness, higher levels of cardiorespiratory fitness were associated with a lower risk of all-cause (top quintile HR: 0.47, 95% confidence interval: 0.46–0.49, *P* < 0.001), cancer (HR: 0.69; 0.65–0.74, *P* < 0.001), and cardiovascular disease mortality (HR: 0.42; 0.39–0.45, *P* < 0.001) (*Figure 1*). In the negative control outcome analysis examining accidental mortality, the association was of similar magnitude (HR: 0.47; 0.44–0.50, *P* < 0.001). In sibling-comparison analysis, even though all estimates attenuated, the comparably lower risk of accidental mortality vs. all-cause, cancer, and cardiovascular disease mortality remained (*Figure 1*). The findings were consistent when accounting for competing risk of death (see Supplementary material online, *Table S4*).

**Figure 1:**
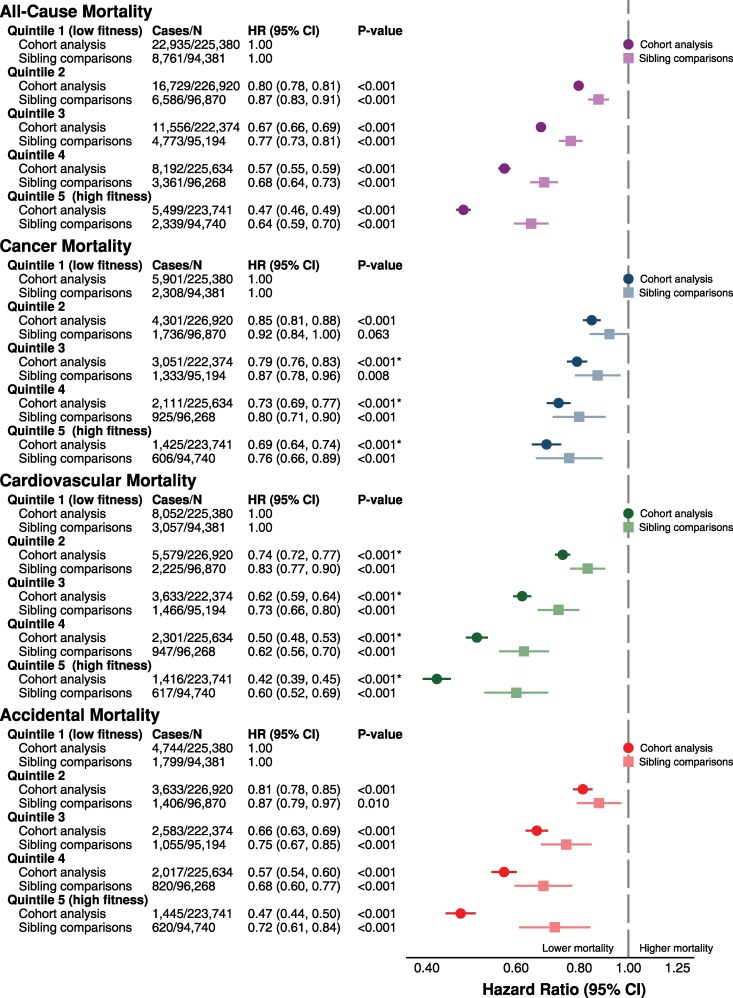
Hazard ratios for all-cause, cancer, cardiovascular disease, and accidental mortality by quintiles of cardiorespiratory fitness in cohort (strong colour) and sibling-comparison analysis (lighter colour). Estimates were obtained using flexible parametric survival models, extended to a marginalized between-within model among full siblings, with baseline knots placed at the 5th, 27.5th, 50th, 72.5th, and 95th percentile of the uncensored log survival times, and using age as the underlying time scale. The lowest quintile (those with the lowest 20% of cardiorespiratory fitness) was the referent. Statistically significant different estimates (compared to accidental mortality estimates) are indicated by an asterisk. All models were adjusted for age at conscription (continuous), year of conscription (six categories) measured body mass index (continuous and quadratic), parental highest level of attained lifetime education (four categories), and parental highest-achieved quintile of birth-year standardized income between ages 40 and 50 (five categories).

In this study of more than 1 million Swedish men spanning six decades, higher levels of cardiorespiratory fitness in late adolescence were strongly associated with lower premature all-cause, cancer, and cardiovascular disease mortality. However, a similarly strong association was observed with accidental mortality, and not even adjustment for unobserved familial confounders shared between full siblings appeared to resolve this bias. This suggests that fitness levels and mortality risk are influenced by strong socioeconomic, genetic, and behavioural confounding factors that differ between more fit and less fit people. Indeed, there is a well-known social gradient in both fitness and major somatic diseases (e.g. cardiovascular disease),^13,14^ as well as a clear contribution of genetic predisposition.^3,15^ Seemingly, these factors are difficult to address even with measured data on education and income, and despite control for all factors shared between siblings. Although one could perhaps conceive of some possible mechanisms causally linking higher fitness with lower accidental mortality (e.g. greater reaction speed when facing difficult situations), we deem this explanation unlikely, as the magnitude of association was similar to that of cardiovascular disease and cancer. We would have expected larger associations with cardiovascular disease and cancer mortality than accidents, given the extensive epidemiological and mechanistic evidence supporting the relationships with these major somatic disease groups.^1^

Study limitations include the exclusion of female participants and that participants were followed only until their 60 s on average. On the other hand, the use of objective fitness measures and nationwide registers with high precision and virtually no attrition strengthens our findings.

In summary, using random non-causal outcomes and sibling-comparisons, we find that observational estimates supporting the role of adolescent cardiorespiratory fitness in preventing premature mortality may be characterized by widespread bias. By implication, the frequently observed lower mortality in highly fit individuals may not reflect a causal relationship to such an extent that is typically assumed, which call into question the claims about the importance of intervening on adolescent cardiorespiratory fitness for reducing disease burden and promoting longevity. Further research using causal analyses with unrelated key sources of bias is warranted to triangulate these findings.^16^ This could include cross-country comparisons (with different bias structures), negative controls, sibling and twin-comparisons, and various types of instrumental variable analyses, including (but not limited to) Mendelian randomization.

## Supplementary Material

zwaf267_Supplementary_Data

## Data Availability

The data in this study are not available to the public and will not be shared according to regulations under Swedish law. Researchers interested in obtaining the data may seek ethical approvals and inquire through Statistics Sweden. For further advice, see https://www.scb.se/en/services/ordering-data-and-statistics/.
