# Supplementary material for: Cardiorespiratory fitness in adolescence and premature mortality: widespread bias identified using negative control outcomes and sibling comparisons
Source: Eur J Prev Cardiol. 2025 May 15;33(9):1715–8. doi: 10.1093/eurjpc/zwaf267 (PMC13364046; doi:10.1093/eurjpc/zwaf267)
Supplement: zwaf267_Supplementary_Data [file zwaf267_supplementary_data.docx]

**Supplemental material**

**Cardiorespiratory fitness in adolescence and premature mortality – widespread bias identified using negative control outcomes and sibling-comparisons**

**Authors:** Marcel Ballin, PhD^1^, Anna Nordström, MD, PhD^2,3^, Peter Nordström, MD, PhD^1^, Viktor H. Ahlqvist, PhD^1,4,5^

**Affiliations:** ^1^Department of Public Health and Caring Sciences, Clinical Geriatrics, Uppsala University, Uppsala, Sweden. ^2^Department of Medical Sciences, Rehabilitation Medicine, Uppsala University, Uppsala, Sweden. ^3^School of Sports Science, UiT The Arctic University of Norway, Tromsø, Norway. ^4^Department of Biomedicine, Aarhus University, Aarhus, Denmark. ^5^Institute of Environmental Medicine, Karolinska Institutet, Stockholm, Sweden.

**Correspondence**: Marcel Ballin. Department of Public Health and Caring Sciences, Clinical Geriatrics, Uppsala University, SE-75103, Uppsala, Sweden. [marcel.ballin@uu.se](mailto:marcel.ballin@uu.se)

**Running head:** Physical fitness, mortality, and residual bias

**Funding:** The author(s) received no specific funding for this work. VHA is funded via grants from the National Institute for Aging and the National Institute of Neurological Disorders and Stroke (1R01NS131433-01). The funders had no role in the design and conduct of the study; collection, management, analysis, and interpretation of the data; preparation, review, or approval of the manuscript; and decision to submit the manuscript for publication.

**Conflict of interest disclosures:** MB is employed at the Swedish Medical Products Agency, SE-751 03 Uppsala, Sweden. The views expressed in this paper do not necessarily represent the views of this Government agency. The authors report no other disclosures.

| **Supplemental table 1. Diagnostic codes used to define mortality from different causes in the study** | | |
| --- | --- | --- |
| **Outcome** | **Code Type** | **Codes** |
| Cancer mortality | ICD-10 | C00-C97 |
|  | ICD-9 | 140-175, 179-208 |
|  | ICD-8 | 140-209 |
| Cardiovascular disease mortality | ICD-10 | I00-I99 |
|  | ICD-9 | 390-459 |
|  | ICD-8 | 390-458 |
| Accidental mortality | ICD-10 | V01-X59* |
|  | ICD-9 | 800-999 |
|  | ICD-8 | 800-999 |
| ICD = International Classification of Diseases. *Excluding V10-19 and X60-84. | | |

| **Supplemental table 2. Baseline characteristics in the total cohort and in the sibling cohort.** | | |
| --- | --- | --- |
|  | **Total cohort (N=1 124 049)** | **Sibling cohort (N=477 453)** |
| **Birth year, median (IQR)** | 1966 (1960 to 1971) | 1965 (1961 to1970) |
| **Age at conscription, mean (SD)** | 18.3 (0.7) | 18.3 (0.7) |
| **Body mass index categories, n (%)** |  |  |
| Underweight (<18.5 kg/m^2^) | 88 966 (7.9) | 37 884 (7.9) |
| Normal weight (18.5-24.9 kg/m^2^) | 913 586 (81.3) | 390 811 (81.9) |
| Overweight (25.0-29.9 kg/m^2^) | 102 267 (9.1) | 41 248 (8.6) |
| Obesity (>30.0 kg/m^2^) | 19 230 (1.7) | 6312 (1.3) |
| **Cardiorespiratory fitness, Wmax, by quintiles, median (range)** |  |  |
| Quintile 1 | 211 (100 to 229) | 211 (100 to 229) |
| Quintile 2 | 243 (230 to 257) | 243 (230 to 257) |
| Quintile 3 | 271 (258 to 286) | 271 (258 to 286) |
| Quintile 4 | 302 (287 to 323) | 302 (287 to 323) |
| Quintile 5 | 345 (324 to 999) | 346 (324 to 999) |
| **Parental level of education, n (%)** |  |  |
| Compulsory school <9 years | 319 242 (28.4) | 139 246 (29.2) |
| Secondary education | 505 510 (45.0) | 210 246 (44.0) |
| Post-secondary education <3 years | 123 757 (11.0) | 50 210 (10.5) |
| Post-secondary education >3 years | 175 540 (15.6) | 77 751 (16.3) |
| **Parental highest income, n (%)** |  |  |
| Category 1 (low income) | 55 055 (4.9) | 18 918 (4.0) |
| Category 2 | 108 836 (9.7) | 41 820 (8.8) |
| Category 3 | 237 953 (21.2) | 101 150 (21.2) |
| Category 4 | 338 444 (30.1) | 145 735 (30.5) |
| Category 5 (high income) | 383 761 (34.1) | 169 830 (35.6) |
| IQR = interquartile range. SD = standard deviation. | | |

| **Supplemental table 3. Follow-up time, number of events, and numbers censored in cohort and sibling analysis.** | | |
| --- | --- | --- |
| **Mortality outcome** | **Cohort analysis**   **(N=1 124 049)** | **Sibling analysis**   **(N=477 453)** |
| **All-cause mortality** |  |  |
| Age at death, median (range) | 50.8 (17.7-72.9) | 50.9 (17.7-72.9) |
| Death | 64 911 (5.8) | 25 820 (5.4) |
| Emigration | 76 274 (6.8) | 31 377 (6.6) |
| End of follow-up | 982 864 (87.4) | 420 256 (88.0) |
| **Cancer mortality** |  |  |
| Age at cancer-related death, median (range) | 55.7 (18.4-72.9) | 55.3 (18.4-72.9) |
| Cancer-related death | 16 789 (1.5) | 6908 (1.5) |
| Death from other causes | 48 122 (4.3) | 18 912 (4.0) |
| Emigration | 76 274 (6.8) | 31 377 (6.6) |
| End of follow-up | 982 864 (87.4) | 420 256 (88.0) |
| **Cardiovascular mortality** |  |  |
| Age at cardiovascular-related death, median (range) | 55.4 (18.1-72.9) | 55.1 (18.2-72.9) |
| Cardiovascular-related death | 20 981 (1.9) | 8312 (1.7) |
| Death from other causes | 43 930 (3.9) | 17 508 (3.7) |
| Emigration | 76 274 (6.8) | 31 377 (6.6) |
| End of follow-up | 982 864 (87.4) | 420 256 (88.0) |
| **Accidental mortality** |  |  |
| Age at accident-related death, median (range) | 32.1 (17.7-70.8) | 32.3 (17.7-70.6) |
| Accident-related death | 14 422 (1.3) | 5700 (1.2) |
| Death from other causes | 50 489 (4.5) | 20 120 (4.2) |
| Emigration | 76 274 (6.8) | 31 377 (6.6) |
| End of follow-up | 982 864 (87.4) | 420 256 (88.0) |
| Number of events and numbers censored are shown as n (%). | | |

| **Supplemental table 4.** **Absolute and relative risks for cancer, cardiovascular disease, and accidental mortality by 65 years of age, without accounting for competing risk of death (net risk) vs accounting for competing risk of death (crude risk), by quintiles of cardiorespiratory fitness in cohort and sibling analysis.** | | | | | | | | |
| --- | --- | --- | --- | --- | --- | --- | --- | --- |
| **Mortality outcome by quintiles of fitness** | **Cohort analysis (N=1 124 049)^a^** | | | | **Sibling analysis (N=477 453)^a^** | | | |
|  | **Net risk^b^** | | **Crude risk^c^** | | **Net risk^b^** | | **Crude risk^c^** | |
|  | **Risk at age 65 y,  % (95% CI)** | **Risk ratio  (95% CI)** | **Risk at age 65 y,  % (95% CI)** | **Risk ratio  (95% CI)** | **Risk at age 65 y,  % (95% CI)** | **Risk ratio  (95% CI)** | **Risk at age 65 y,  % (95% CI)** | **Risk ratio  (95% CI)** |
| **Cancer mortality** | | | | | | | | |
| Quintile 1 | 3.06 (2.95-3.17) | Ref. | 2.83 (2.73-2.94) | Ref. | 2.82 (2.62-3.05) | Ref. | 2.66 (2.46-2.87) | Ref. |
| Quintile 2 | 2.60 (2.50-2.69) | 0.85 (0.82-0.88) | 2.45 (2.36-2.54) | 0.86 (0.83-0.90) | 2.59 (2.41-2.79) | 0.92 (0.84-1.00) | 2.46 (2.29-2.65) | 0.93 (0.86-1.01) |
| Quintile 3 | 2.43 (2.34-2.53) | 0.79 (0.76-0.83) | 2.31 (2.23-2.41) | 0.82 (0.78-0.85) | 2.46 (2.28-2.65) | 0.87 (0.79-0.97) | 2.35 (2.18-2.53) | 0.89 (0.80-0.98) |
| Quintile 4 | 2.24 (2.14-2.34) | 0.73 (0.69-0.77) | 2.15 (2.06-2.25) | 0.76 (0.72-0.80) | 2.27 (2.08-2.47) | 0.80 (0.71-0.90) | 2.18 (2.00-2.37) | 0.82 (0.73-0.92) |
| Quintile 5 | 2.12 (2.01-2.24) | 0.69 (0.65-0.74) | 2.05 (1.94-2.17) | 0.72 (0.68-0.77) | 2.16 (1.94-2.42) | 0.77 (0.66-0.89) | 2.08 (1.87-2.33) | 0.79 (0.68-0.91) |
| **Cardiovascular mortality** | | | | | | | | |
| Quintile 1 | 4.52 (4.38-4.66) | Ref. | 4.24 (4.11-4.37) | Ref. | 3.67 (3.43-3.92) | Ref. | 3.47 (3.25-3.71) | Ref. |
| Quintile 2 | 3.38 (3.27-3.49) | 0.75 (0.72-0.77) | 3.20 (3.10-3.31) | 0.76 (0.73-0.78) | 3.06 (2.87-3.26) | 0.83 (0.77-0.90) | 2.91 (2.73-3.10) | 0.84 (0.78-0.91) |
| Quintile 3 | 2.82 (2.72-2.92) | 0.62 (0.60-0.65) | 2.69 (2.60-2.79) | 0.64 (0.61-0.66) | 2.69 (2.51-2.88) | 0.73 (0.67-0.80) | 2.57 (2.40-2.76) | 0.74 (0.68-0.81) |
| Quintile 4 | 2.30 (2.21-2.40) | 0.51 (0.49-0.54) | 2.21 (2.12-2.31) | 0.52 (0.50-0.55) | 2.30 (2.12-2.50) | 0.63 (0.56-0.70) | 2.21 (2.04-2.40) | 0.64 (0.57-0.71) |
| Quintile 5 | 1.92 (1.82-2.03) | 0.43 (0.40-0.45) | 1.86 (1.77-1.97) | 0.44 (0.41-0.47) | 2.22 (2.00-2.48) | 0.61 (0.53-0.70) | 2.14 (1.92-2.39) | 0.62 (0.54-0.71) |
| **Accidental mortality** | | | | | | | | |
| Quintile 1 | 2.57 (2.42-2.74) | Ref. | 2.53 (2.38-2.70) | Ref. | 2.09 (1.88-2.32) | Ref. | 2.06 (1.86-2.29) | Ref. |
| Quintile 2 | 2.10 (1.07-2.23) | 0.82 (0.78-0.85) | 2.07 (1.95-2.21) | 0.82 (0.78-0.85) | 1.83 (1.65-2.03) | 0.88 (0.79-0.97) | 1.81 (1.63-2.00) | 0.88 (0.79-0.97) |
| Quintile 3 | 1.70 (1.60-1.82) | 0.66 (0.63-0.70) | 1.69 (1.58-1.80) | 0.67 (0.63-0.70) | 1.58 (1.42-1.76) | 0.76 (0.67-0.85) | 1.56 (1.41-1.74) | 0.76 (0.68-0.85) |
| Quintile 4 | 1.47 (1.37-1.58) | 0.57 (0.54-0.61) | 1.46 (1.36-1.56) | 0.58 (0.54-0.61) | 1.43 (1.27-1.60) | 0.68 (0.60-0.78) | 1.41 (1.26-1.59) | 0.68 (0.60-0.78) |
| Quintile 5 | 1.21 (1.12-1.30) | 0.47 (0.44-0.50) | 1.20 (1.11-1.30) | 0.47 (0.44-0.51) | 1.50 (1.31-1.72) | 0.72 (0.61-0.84) | 1.49 (1.29-1.71) | 0.72 (0.62-0.85) |
| ^a^The flexible parametric models were performed in the full sample, from which the standardised incidences were computed in a random subsample of 10%.  ^b^Standardised cumulative incidences as obtained in the main analysis, assuming conditional independence between time to the primary outcome and the competing event (death from other causes).  ^c^Cause-specific standardised cumulative incidence functions, accounting for the competing risk of death from other causes.  CI = confidence interval. All estimates are adjusted for age at conscription, year of conscription, body mass index, parental education, and parental income. | | | | | | | | |
